# A Novel Europium Chelate Coated Nanosphere for Time-Resolved Fluorescence Immunoassay

**DOI:** 10.1371/journal.pone.0129689

**Published:** 2015-06-09

**Authors:** Yifeng Shen, Shaohan Xu, Donghua He

**Affiliations:** 1 Key Laboratory of Ministry of Education for Gastrointestinal Cancer, School of Basic Medical Sciences, Fujian Medical University, Fuzhou, Fujian, China; 2 The First Affiliate Hospital of Fujian Medical University, Fuzhou, Fujian, China; 3 Triplex International Bioscience (China) CO., Ltd., Xiamen, Fujian, China; CNR, ITALY

## Abstract

A novel europium ligand 2, 2’, 2’’, 2’’’-(4, 7-diphenyl-1, 10-phenanthroline-2, 9-diyl) bis (methylene) bis (azanetriyl) tetra acetic acid (BC-EDTA) was synthesized and characterized. It shows an emission spectrum peak at 610 nm when it is excited at 360 nm, with a large Stock shift (250 nm). It is covalently coated on the surface of a bare silica nanosphere containi free amino groups, using 1-ethyl-3-(3-dimethylaminopropyl) carbodiimide hydrochloride and N-Hydroxysuccinimide. We also observed an interesting phenomenon that when BC-EDTA is labeled with a silica nanosphere, the chelate shows different excitation spectrum peaks of about 295 nm. We speculate that the carboxyl has a significant influence on its excitation spectrum. The BC-EDTA/Eu^3+^coated nanosphere could be used as a fluorescent probe for time-resolved fluorescence immunoassay. We labeled the antibody with the fluorescent nanosphere to develop a nanosphere based hepatitis B surface antigen as a time-resolved fluorescence immunoassay reagent, which is very easy to operate and eliminates potential contamination of Eu^3+^ contained in the environment. The analytical and functional sensitivities are 0.0037 μg/L and 0.08 μg/L (S/N≥2.0) respectively. The detection range is 0.08-166.67 μg/L, which is much wider than that of ELISA (0.2-5μg/L). It is comparable to the commercial dissociation-enhanced lanthanide fluoro-immunoassay system (DELFIA) reagents (0.2-145μg/L). We propose that it can fulfill clinical applications.

## Introduction

Multiple studies reported that lanthanide and its chelate can be applied in time-resolved fluorescence immunoassay (TrFIA)[1, 2]. It is widely used in clinical immunoassay, such as in DELFIA reagents (Perkinelmer Inc.). Europium and its chelate have many features that make them suitable for TrFIA, the maximum excitation wavelength of Eu^3+^ fluorescent complexes is in the UV region and the emission maximum is about 610 nm, which is ion-specific. This large Stokes shift can avoid the interference of excitation light. The emission band of Eu^3+^ fluorescent complexes is very narrow, with the full width at half maximum (FWHM) being about 10 nm. The most important feature of the complexes is they have very long fluorescence lifetimes ranging from 1 μs to over 2 ms, which are longer than background fluorescence. These characteristics of Eu^3+^ fluorescent complexes can be used to detect biomolecules using time-resolved fluorometry with high sensitivity[3].

Dual-functional europium chelate isothiocyanatophenyl-EDTA-Eu^3+^ and N^1^-(p-isothiocyanatobenzyl) diethylenetriamine-N^1^, N^2^, N^3^, N^4^—tetra acetic acid-Eu^3+^ is used in the dissociation-enhanced lanthanide fluoroimmunoassay system (DELFIA), which has been commercially available for decades. These chelates are not fluorescent; they only can bind Eu^3+^ with antibodies. After immunological reaction, Eu^3+^ dissociates in acid buffer and combines with other fluorescent ligands, such as β-naphthoyltrifluoroacetone (β-NTA), resulting in fluorescent complexes that emit strong fluorescence[4]. Although the DELFIA system has a high sensitivity, it is vulnerable to Eu^3+^ contamination. This is because the excess ligands can be contaminated by the Eu^3+^ contained in the environment leading to a high background level. Furthermore, the fluorescent signal is not bound to the antibody directly[4, 5].

In the CyberFluor system, europium ligand 4, 7-bis (chlorosulfophenyl) -1, 10-phenanthroline-2, 9-dicarboxylic acid (BCPDA) was directly labeled with protein[6]. In this system, the background is low because no excess ligands exist in the solution. However because BCPDA has only 4 chelate sites, the fluorescence of the chelate is weaker than the β-NTA-Eu^3+^ complex contained in the DELFIA system[7]. When BBCAP chelates with Eu^3+^, it has an emission maximum at 610 nm when excited with UV light of 280 nm[8]. Jingli Yuan and Kazuko Matsumoto first synthesized 4, 4-bis (1, 1, 2, 2, 3, 3-heptafluoro-4, 6-hexanedion-6-yl) chlorosulfo-ο-terphenyl (BHHCT), which is a kind of tetradentate β-diketonate-europium ligand. It shows an emission at 610 nm when excited at 330 nm[9]. The usage of BHHCT is limited because it can only dissolve in organic solutions and it has poor solubility in H_2_O[9]. Peng Lin et al. synthesized BBCAP a bis-functional ligand, and applied it in TrFIA[10]. These ligands indicate that phenanthroline is suitable for efficient transfer of energy from the ligand to Eu^3+^ with a high fluorescence quantum yield.

Many studies reported that using a multiple-label system is an effective approach to amplifying signals of existing chelates[11–13]. In multiple label systems, streptavidin[7] and polymeric compounds[11, 14] are used as the molecular carriers. The great number of fluorescent chelates carried by a single carrier can serve as a probe to conjugate with an antibody for TrFIA, yielding a high sensitivity. A polystyrene nanosphere contains europium chelates synthesized as a probe for TrFIA, which can provide a very high sensitivity. The polystyrene nanosphere was conjugated with streptavidin or antibodies to detect prostate-specific antigen[15, 16], and thyroid stimulating hormone[17] in an easy-to-operate system that provided very high sensitivity. A silica nanosphere which contains Eu^3+^ or Tb^3+^ chelates is another choice for a TrFIA probe[18–20]. Silica nanospheres containing europium and terbium chelates were easily synthesized; fluorescence chelates can be physically doped or covalently conjugated with a silica nanosphere. These nanospheres containing europium chelates with multiple chelate sites contributed to high sensitivity TrFIA reagents. Furthermore, because there are no surplus ligands, its background is much lower than that of DELIFA reagents.

**Fig 1:**
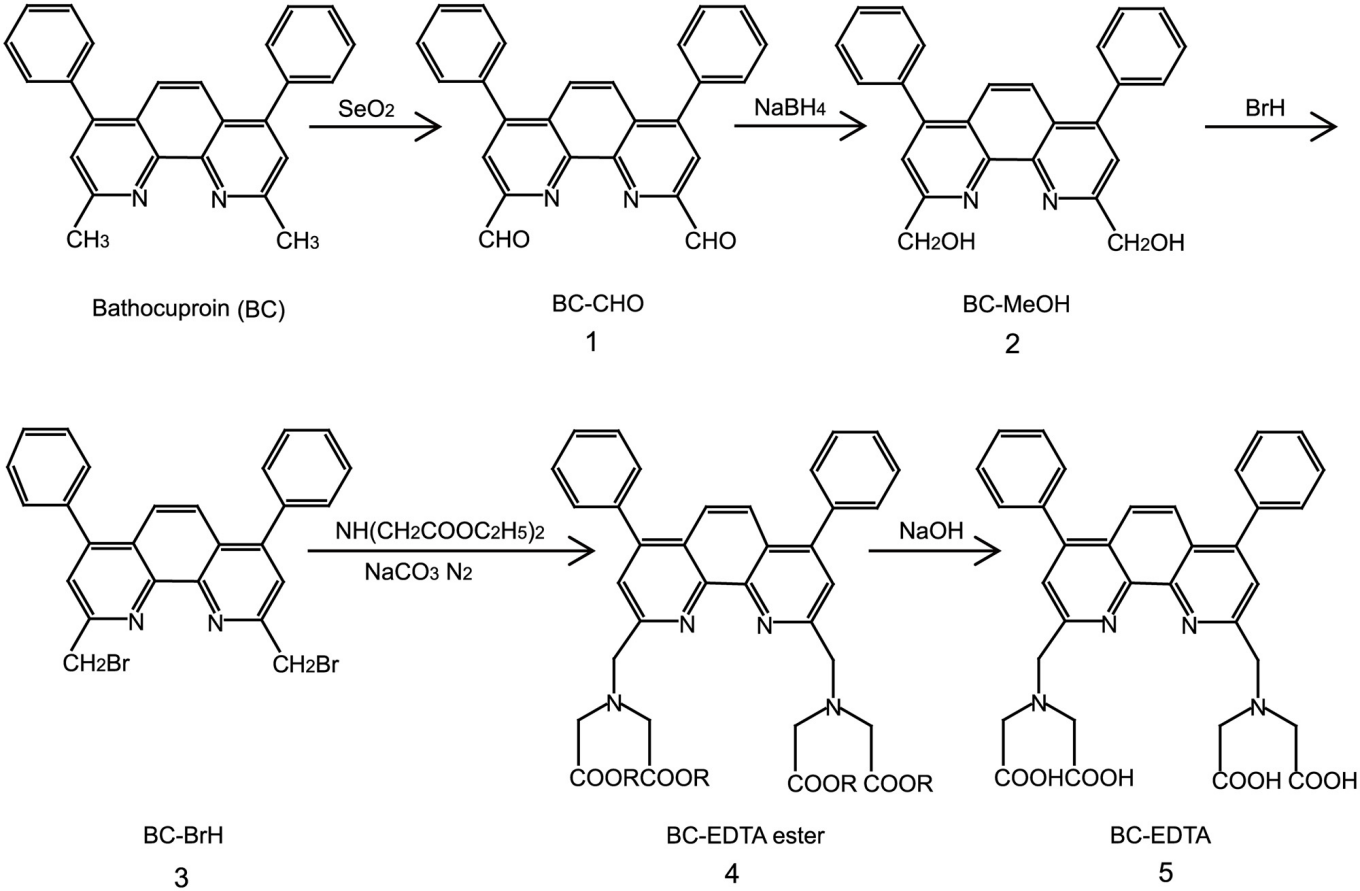
Synthesis of BC-EDTA.

In this paper, we report that a novel europium ligand contains 8 chelate sites, namely 2, 2’, 2”, 2”‘- (4, 7-diphenyl-1, 10-phenanthroline-2, 9-diyl) bis (methylene) bis (azanetriyl) tetra acetic acid (BC-EDTA,Fig 1). It forms a stable complex with Eu^3+^, and emits strong fluorescence when excited with UV light, at 360 nm. It can be easily dissolved in H_2_O. It was covalently coated onto the surface of a preformed bare silica nanosphere with free amino groups, resulting in a fluorescent nanosphere. Biomarkers such as antibodies can be labeled with such nanospheres to form probes. We developed a nanosphere based time-resolved fluorescence immunoassay (TrFIA) to detect surface antigen of hepatitis B virus (HBsAg), with the analytical and functional sensitivities at 0.0037 μg/L and 0.08 μg/L (S/N≥2.0), respectively. The detection range was 0.08–166.67 μg/L, which is much wider than ELISA (0.2–5μg/L), and is comparable to commercial dissociation-enhanced lanthanide fluoroimmunoassay system (DELFIA) reagents. Additionally, the nanosphere based TrFIA is much easier to manipulate than DELFIA reagents and eliminates potential Eu^3+^ contamination.

## Methods

### Synthesis of BC-EDTA

BC-EDTA was synthesized according to the following scheme (Fig 1), the synthesis procedure was illuminated by the synthesis of BCPDA[6] and BBCAP[10]. Ethanol and acetonitrile must be distilled to remove the surplus water.

#### (1) Materials and Instruments

1, 4-dioxane aqueous, kieselguhr, anhydrous ethanol, anhydrous acetonitrile, sodium carbonate, acetone, sodium hydroxide, and hydrochloric acid were purchased from SCRC. Bathocuproine (BC), selenium dioxide, sodium borohydride, and hydrobromic acid was purchased from ABCR. Diethyl iminodiacetat was purchased from Sigma. Anhydrous ethanol was distilled from magnesium and iodine. Anhydrous acetonitrile was distilled from phosphorus pentoxide.

^1^H NMR spectra were recorded on Varian Unity+ 500M Hz. ^1^H NMR spectra were registered in D_2_O, and chemical shifts are expressed in partsper million (δ) relative to internal Me_4_Si. IR spectra were recorded on a Nicolet Avatar 330 FTIR spectrophotometer. Mass spectra were recorded by Bruke BioTOF-Q. UV spectra were recorded on a Persee TU-1900 ultraviolet and visible spectrophotometer.

#### (2) Preparation of BC-CHO

Bathocuproin (BC) (0.5 g, 1.39mmol) and selenium dioxide (0.75 g, 6.76 mmol) were gradually added to 50 mL stirring 1, 4-dioxane aqueous solution (4% H_2_O) and the mixture was refluxed for 2 hours at 101°C. The mixture was filtered by kieselguhr while still hot and then mixed with water and stored at 2–8°C for 1 hour. The precipitate was separated from the cold filtrate as brown crystals with a yield of 79.8% (0.43 g). MS (ESI): 389.2 m/z (M+H^+^).

#### (3) Preparation of BC-MeOH

BC-CHO (0.4 g, 1.03 mmol) and aqueous sodium borohydride (0.06 g, 1.59 mmol) were gradually added to 40 mL stirring anhydrous ethanol and refluxed for 2 hours at 100°C. The mixture was then filtered, and the resulting filtrate was mixed with water and stored at 2–8°C for 1 hour. The precipitate was separated from the cold filtrate as a pink powder with a yield of 82.7% (0.45 g). MS (ESI): 415.5 m/z (M+Na^+^).

#### (4) Preparation of BC-BrCH_2_

BC-MeOH (0.4 g, 1.02 mmol) was added to 12 mL hydrobromic acid (48%) and refluxed for 2 hours. The mixture was cooled in ice water and then aqueous sodium carbonate was gradually added until the precipitate was fully formed. 30 mL ethanol was then added and the mixture was placed in a boiling water bath to completely dissolve the precipitate. The filtrate was collected and mixed with water, then stored at 2~8°C for 1 hour. A yellow powder of BC-BrCH_2_ was then collected and dried with a yield of 45.9% (0.33 g). MS (ESI): 519.2 m/z (M+H^+^).

#### (5) Preparation of BC-EDTA ester

BC-BrCH_2_ (0.3 g, 0.58 mmol) and sodium carbonate (0.45 g, 4.25 mmol) were combined in stirring 20 mL anhydrous acetonitrile and diethyl iminodiacetate (210 μL, 1.17 mmol) was gradually added. Then the mixture was stirred in darkness in a nitrogenous atmosphere to avoid photobleaching and oxidation. This reaction took place at room temperature for 24 hours. This filtrate was then collected and vacuum-dried. A brown solid of BC-EDTA ester was obtained with a yield of 53.97% (0.55 g). MS (ESI): 758.1 m/z (M+Na^+^).

#### (6) Preparation of BC-EDTA

**Fig 2:**
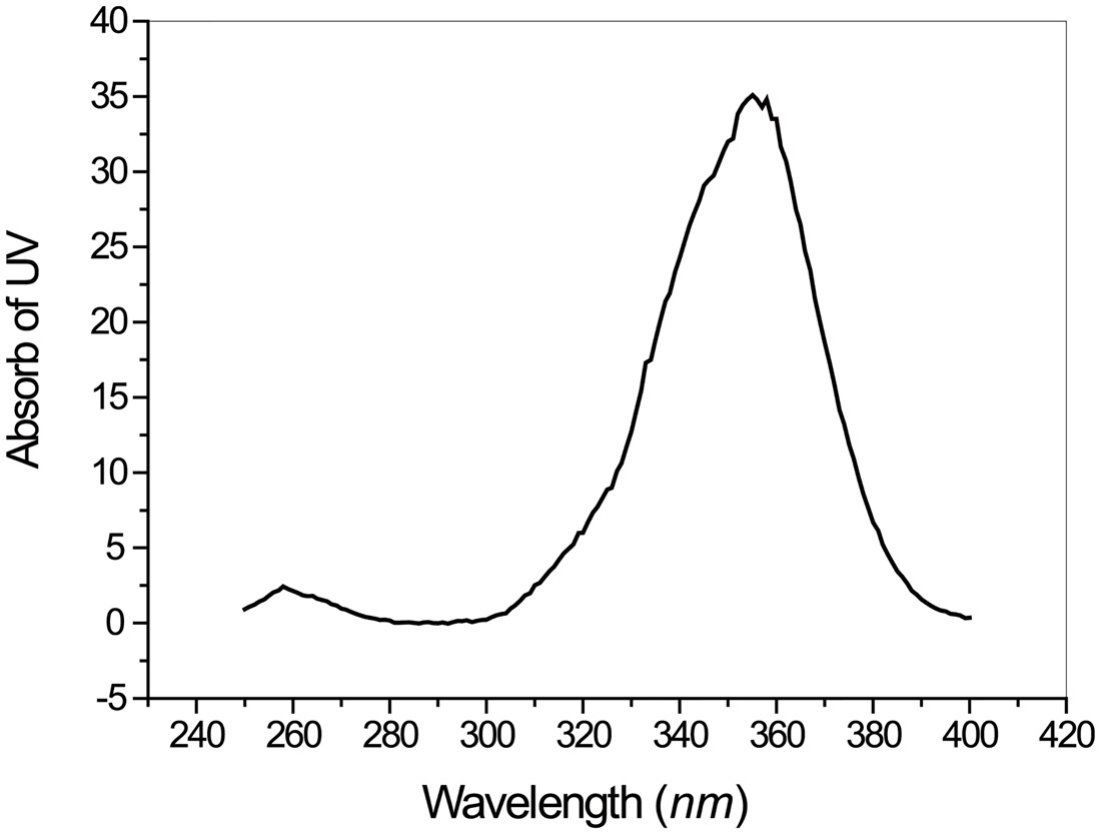
Ultraviolet-Visible (UV) Absorption Spectrum of BC-EDTA.

4 mL sodium hydroxide solution (1 mol/L) was gradually added to a solution of 0.5 g (0.68 mmol) BC-EDTA ester and 8 mL acetone. After stirring for 2 hours in darkness to avoid photobleaching, the mixture was neutralized with hydrochloric acid. The liquid was then vacuum-dried, washed with acetone 3 times, and then recrystallized 3 times from water. A brown powder of BC-EDTA was obtained with a yield of 53.2% (0.46 g). MS (ESI): 623.3 m/z (M+H^+^). IR (film) νmax: 3431, 2922, 1631, 1602, 1496, 1403, 1332, 1263 cm^-1^; ^1^H NMR (500 MHz, D_2_O) δ00 M): 3.295 ppm (s, 8H), 4.094 ppm (s, 4H), 7.356 (m, 14H) ppm; UV spectrum: λmax = 360nm (Fig 2).

### Preparation of Silica Nanosphere and Labeling of BC-EDTA

#### (1) Preparation of Bare Silica Nanosphere

The method of preparation of the nanoscale bare silica nanosphere was described by Bagwe RP, Santra S[21, 22]. Amino groups were introduced to the surface of particles by (3-Aminopropyl) trimethoxysilane (APTMS) [23]. A15 mg bare nanosphere was suspended in 1 mL ethanol, then 3 μL APTMS was added into the suspension, and stirred for 2 hours at room temperature. The nanosphere was washed three times with ethanol, and then was suspended in 1 mL 0.05 mol/L phosphate buffer (pH6.8).

#### (2) Preparation of BC-EDTA/Eu^3+^ conjugated Silica Nanosphere

**Fig 3:**
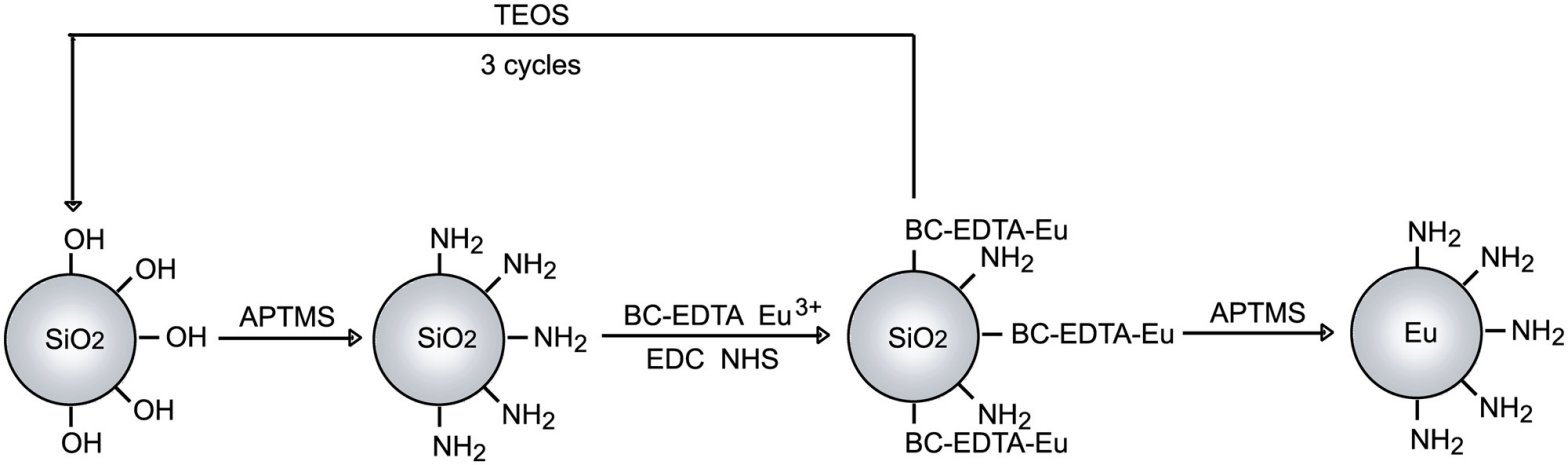
Outline of labeling bare particles with BC-EDTA.

BC-EDTA/Eu^3+^ was conjugated with the silica nanosphere by the following scheme (Fig 3) [24].

BC-EDTA (3.4 mg), 1-ethyl-3-(3-dimethylaminopropyl) carbodiimide hydrochloride (EDC) (3.9 mg), and N-Hydroxysuccinimide (NHS) (0.4 mg) were dissolved in 200 μL 0.05 mmol/L phosphate buffer (pH 6.8), and left to react for 30 minutes in darkness. This solution was then mixed with the nanosphere described above. The nanosphere was washed and suspended in 1 mL 0.01 mol/L Tris-HCl (pH 7.8). 200 μL EuCl_3_ (0.04 mol/L) was added to the nanosphere suspension and left to react for 20 minutes in darkness. It was washed in 1 mL 0.01 mol/L Tris-HCl (pH7.8) 3 times and then suspended in 1 mL ethanol. 20μL tetraethylsilane (TEOS) was added to the suspension, stirred in darkness for 2 hours, and then resuspended in 1mL ethanol. 3 μL APTMS was then added to the solution, stirred in darkness for 2 more hours, then placed in a metal bath at 69°C for 5 minutes and washed in ethanol 3 times.

The procedure was repeated 5 times completely, and the BC-EDTA/Eu^3+^- nanoshpere was suspended and stored in ethanol.

### Labeling of antibody with BC-EDTA/Eu^3+^-nanosphere

**Fig 4:**
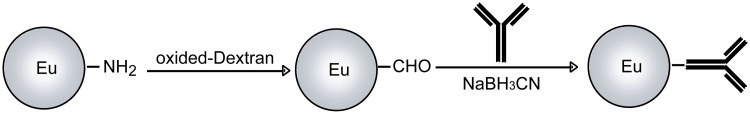
Scheme of labeling of antibody with BC-EDTA/Eu^3+^-nanosphere.

An antibody (anti HBsAg monoclonal antibody IgG, 5#) was conjugated with the BC-EDTA/Eu^3+^ nanosphere by oxided-dextran (Fig 4) [24]. 1 mL 0.5mol/L NaIO_4_ was mixed with 50 mg/mL dextran and left to react at room temperature for 2 hours. The mixture was then dialyzed in ice water. 2 mg BC-EDTA/Eu (III)-nanosphere was washed and suspended in 0.025mol/L carbonate buffer (pH 9.6) and mixed with oxided dextran, left to react in darkness at 37°C for 3 hours, then washed and suspended in 0.025mol/L carbonate buffer (pH 9.6). 0.2 mg antibody was dialyzed in 0.025mol/L carbonate buffer (pH9.6), mixed with the above nanosphere, left to react in darkness at 2~8°C for 12 hours, and then NaBH_3_CN was added to the concentration of 0.005mol/L. BSA (10mg/mL) was used as the blocking agent. The mixture was washed and suspended in 0.01mL Tris-HCl (pH 7.8), and stored in darkness at 2–8°C.

### Unspecific conjugation of antibody labeled BC-EDTA/Eu^3+^-nanosphere

An antibody labeled as BC-EDTA/Eu^3+^-nanosphere was diluted to the concentration of 0.002%, 0.001%, and 0.0005%. The 100μL diluted antibody labeled as BC-EDTA/Eu^3+^-nanosphere was added onto a capture antibody-coated microwell plate (anti HBsAg monoclonal antibody IgG, 2#). The plate was incubated at 37°C for 60 min, and washed 5 times with washing buffer, then it was subjected to TRF measurement (Perkinelmer Victor X).

### Labeling of antibody with BC-EDTA/Eu^3+^-nanosphere

The antibody labeled as BC-EDTA/Eu^3+^-nanosphere was diluted to concentrations of 0.002%, 0.001%, 0.0005%, 0.00025%, and 0.000125%. The 100μL diluted antibody labeled as BC-EDTA/Eu^3+^-nanosphere was added onto an antigen-coated microwell plate (recombinant HBsAg, 0.4μg/mL, 0.2μg/mL). The plate was incubated at 37°C for 60 minutes, and washed 5 times with washing buffer, and then was subjected to TRF measurement (Perkinelmer Victor X).

### Measurement of HBsAg

**Fig 5:**
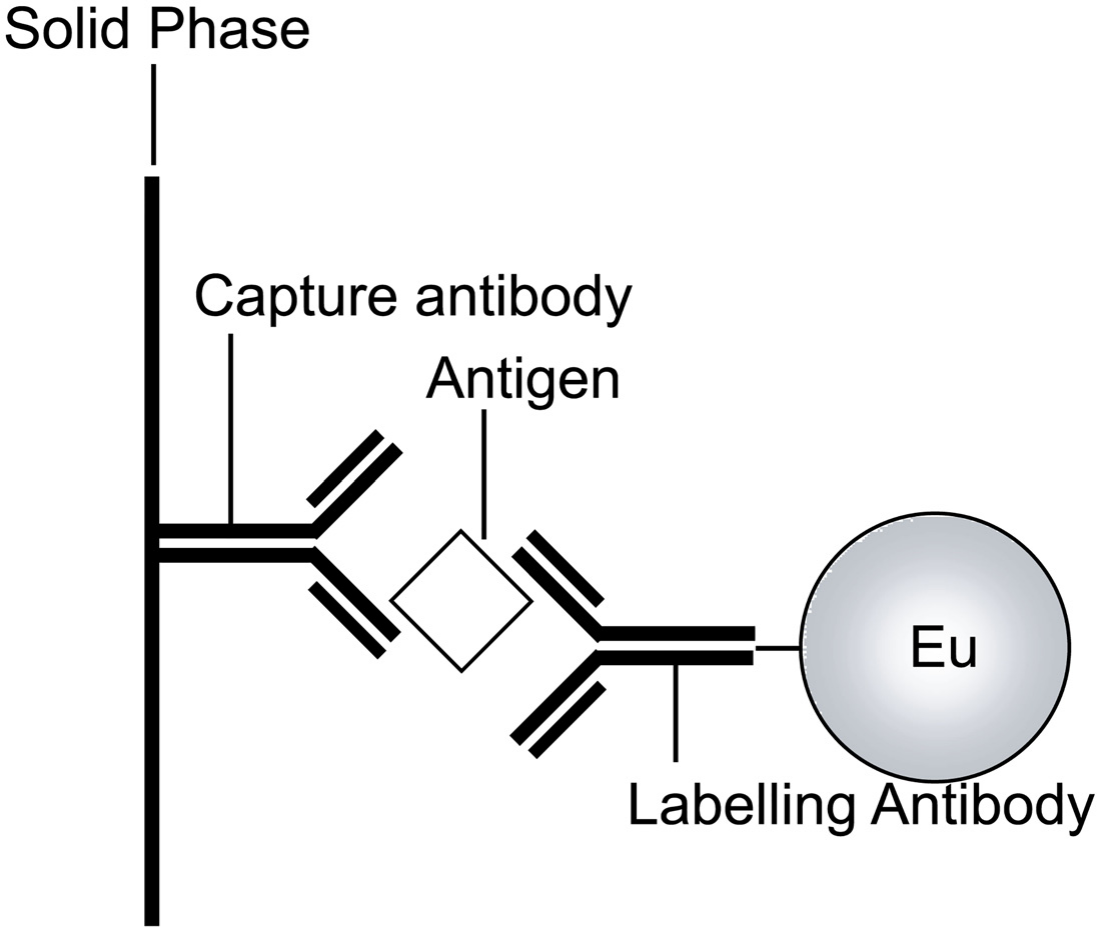
Schematic representation of the immunoassay system for HBsAg by BC-EDTA/Eu^3+^-nanosphere.

A double antibody sandwich method was applied for HBsAg TrIFA (Fig 5).

A capture antibody (anti HBsAg antibody IgG, 2#, 5 μg/mL in 0.02 mol/L phosphate buffer, pH7.4) was physically coated on the white plastic microwell plate (50 μL/well), and the microwell plate was blocked by 2% BSA. 100μL of standard solution for calibration or serum sample was added to each well, and incubated at 37°C for 60 min, then washed 3 times by washing buffer (0.02mol/L phosphate buffer, pH7.4, 0.2% tween-20). Then 100μL of labeling antibody conjugated BC-EDTA/Eu^3+^-nanosphere (diluted 1000-fold) was added to each well. The plate was incubated at 37°C for 60 min, washed 5 times with washing buffer, and then was subjected to TRF measurement (Perkinelmer Victor X).

We still did a contrast test with commercial HBsAg DELFIA reagents (Sym-bio lifescience Inc., China). In total, 230 human serum samples were detected by BC-EDTA/Eu^3+^- nanosphere based TrFIA and DELFIA reagents. A pair T test was used to verify correlation between BC-EDTA/Eu^3+^- nanosphere based TrFIA and DELFIA reagents. We also subjected HBsAg reference panel to BC-EDTA/Eu^3+^- nanosphere based TrFIA for the validation study.

### Human Samples

Human blood samples were collected by vacuum tube from hospital patients, serum was separated by centrifugation. Blood samples used in this study were surplus of those collected for routine clinical tests from clinical laboratories. We did not take special samples for this study.

### Ethics Statement

The study was specifically approved by the Medical Ethics Committee of Fujian Medical University and a written informed consent was signed for each participant.

## Results

### Preparation of BC-EDTA conjugated silica nanosphere

**Fig 6:**
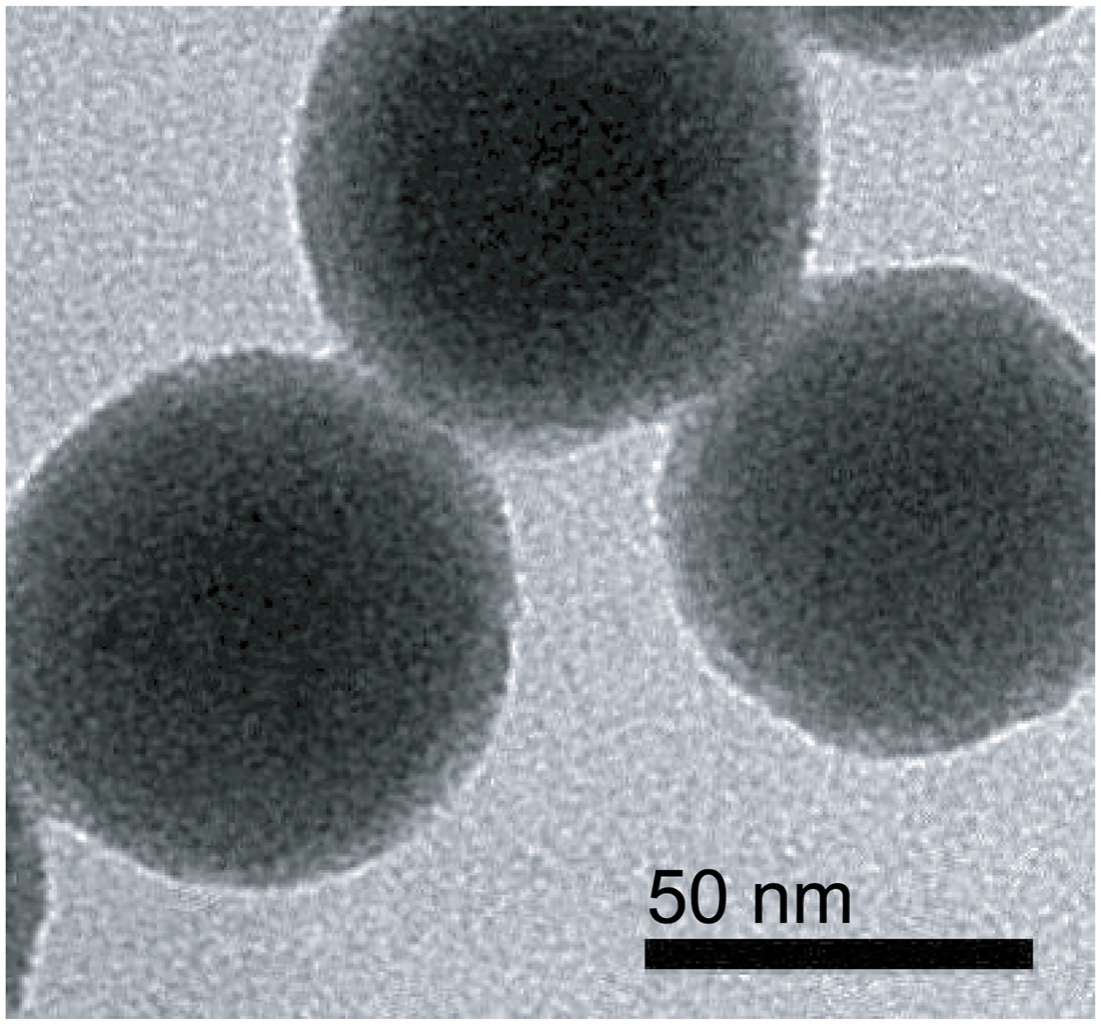
TEM of bare silica nanosphere.

**Fig 7:**
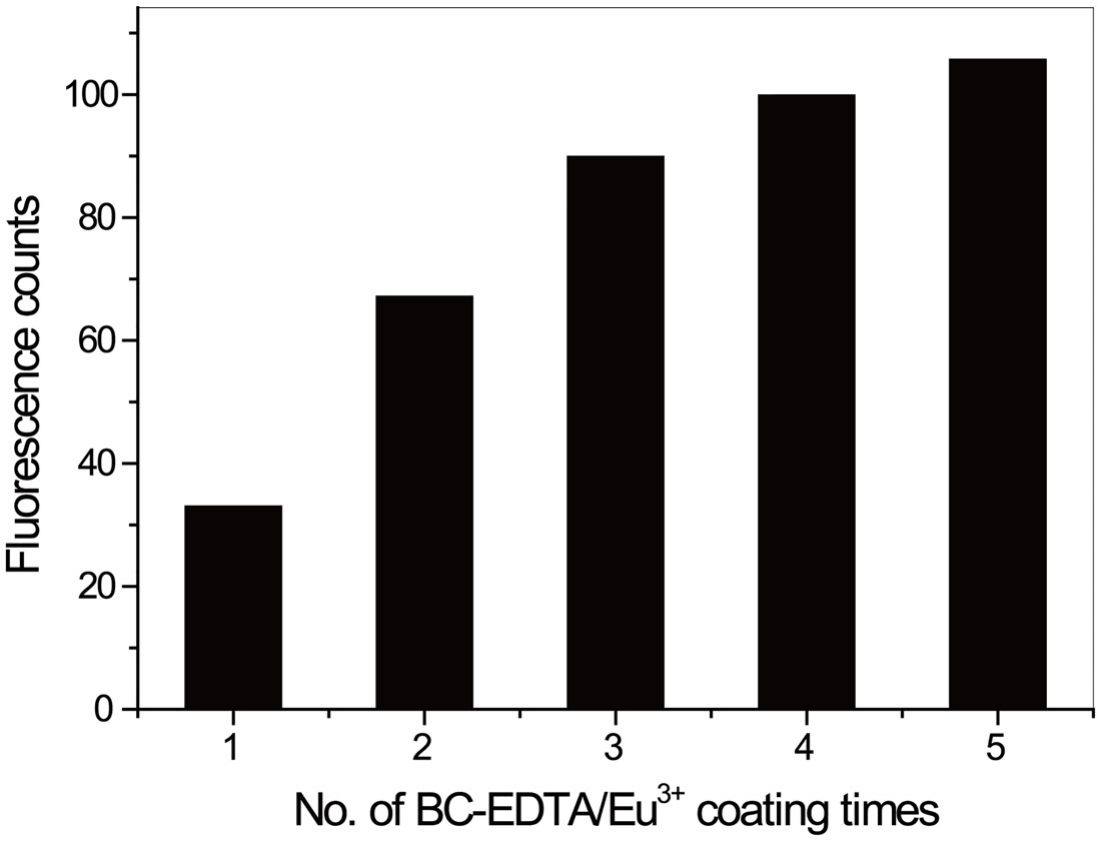
Relationship between fluorescence intensity of nanosphere and coating times. BC-EDTA/Eu^3+^-nanosphere was diluted in 0.05 mol/L carbonate buffer, pH9.5, with the concentration of 0.01%.

Prepared bare silica nanosphere is spherical and uniform in size (55±5nm in diameter, Fig 6). After APTMS treatment, the existence of amino groups on the surface was confirmed by the observation of salicylaldehyde-mediated yellow color (data not shown). Coating was done in multiple rounds to increase the overall chelate loading. With BC-EDTA/Eu^3+^, maximal loading was achieved after 5 rounds of coating (Fig 7). The fluorescent nanosphere showed light aggregation in ethanol, and can be easily dispersed by ultrasonic treatment.

**Fig 8:**
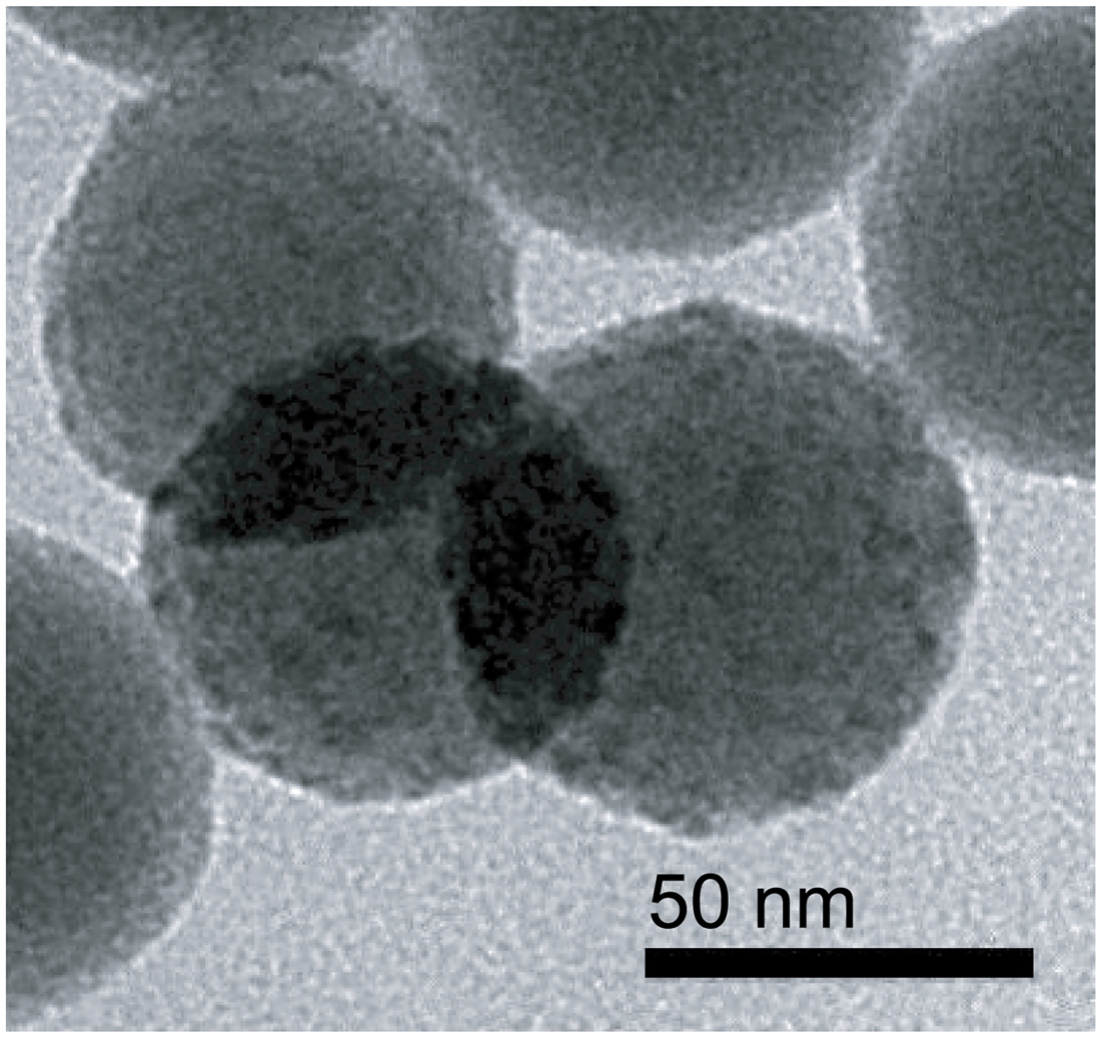
TEM of BC-EDTA/Eu^3+^-nanosphere.

**Fig 9:**
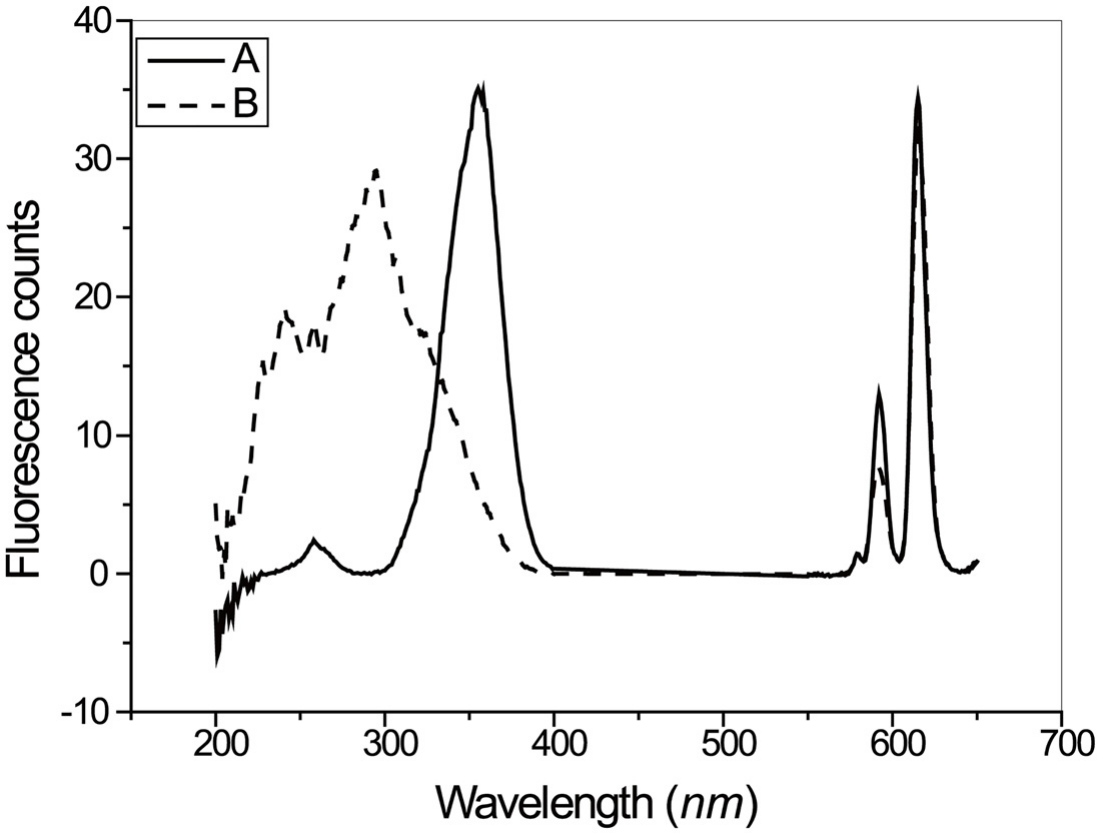
Excitation and Emission spectra of BC-EDTA/Eu^3+^ (A) and BC-EDTA/Eu^3+^-nanosphere (B). (A) The concentration of BC-EDTA/Eu^3+^ is 10^-3^mol/L. The buffer was 0.05 mol/L carbonate buffer, pH9.5. (B) The concentration of BC-EDTA/Eu^3+^-nanosphere is 0.005%. The buffer was 0.05 mol/L carbonate buffer, pH9.5.

The BC-EDTA/Eu^3+^ coated nanosphere (Fig 8) didn’t show significant difference in size and shape compared with the bare silica nanosphere (Fig 6). We found an interesting phenomenon where the maxima excitation wavelength of a BC-EDTA nanosphere is shortened compared with a bare one, and peaks at about 295 nm (Fig 9). We also found that when BC-EDTA reacted with EDC and NHS, its excitation spectrum showed the same difference. It is speculated that carboxyl has an important influence on its excitation. The fluorescence lifetimes of free and coated BC-EDTA/Eu^3+^ were 0.48 and 0.51 ms, respectively.

### Unspecific conjugation of antibody labeled BC-EDTA/Eu^3+^-nanosphere

Overall, the unspecific conjugation rate of an antibody labeled BC-EDTA/Eu^3+^-nanosphere and an unlabeled BC-EDTA/Eu^3+^-nanosphere is less than 0.10% (Table 1). This means that there is no unspecific conjugation between an antibody coated microwell plate and an antibody labeled BC-EDTA/Eu^3+^-nanosphere, neither between the antibody coated microwell plate and the unlabeled BC-EDTA/Eu^3+^-nanosphere.

**Table 1:** Unspecific conjugation of antibody labeled BC-EDTA/Eu^3+^-nanosphere, unlabeled BC-EDTA/Eu^3+^-nanosphere to antibody coated microwell plate.

Nanosphere concentration	antibody labeled BC-EDTA/Eu^3+^- nanosphere			unlabeled BC-EDTA/Eu^3+^- nanosphere		
	Counts^a^	CV (%)	Unspecific conjugation (%)	Counts^a^	CV (%)	Unspecific conjugation (%)
0.002%	310.9	1.94	0.29	311.5	2.01	0.03
0.001%	310.3	2.18	0.03	309.8	1.73	0.06
0.0005%	307.5	2.08	0.10	308.2	2.06	0.06

^a^ Mean value of net fluorescence counts (n = 10).

### Labeling of antibody with BC-EDTA/Eu^3+^-nanosphere

Overall, the specific conjugation rate of an antibody with BC-EDTA/Eu^3+^-nanosphere was no less than 21997.98% (Table 2). This means that the antibody was successfully conjugated with BC-EDTA/Eu^3+^-nanosphere. It further indicates that the antibody still keeps its biological activity, and that the antibody can recognize and binds with HBsAg.

**Table 2:** Specific conjugation of antibody labeled BC-EDTA/Eu^3+^-nanosphere to HBsAg coated mircowell plate.

Nanosphere concentration	0.4μg/mL HBsAg coated mircowell plate			0.2μg/mL HBsAg coated mircowell plate		
	Counts^a^	CV (%)	Specific conjugation (%)	Counts^a^	CV (%)	Specific conjugation (%)
0.002%	15877191	3.30	5138249.51	13157217.6	4.27	4222470.35
0.001%	7498215.8	2.27	2426607.06	6573750.9	2.30	2109676.16
0.0005%	3467750.4	2.42	1122249.32	3137204.4	4.13	1006805.01
0.00025%	1719485	2.50	577963.11	1413288.1	3.93	453558.44
0.000125%	845897.6	2.14	273753.27	68545.7	3.58	21997.98

^a^ Mean value of net fluorescence counts (n = 10).

### Measurement of HBsAg

The analytical sensitivity was 0.0037 μg/L, defined by 3 SD of zero calibrator, functional sensitivity was 0.08 μg/L, which was equivalent to the calibrator with S/N≥2.0 (Table 3). The analytical precision of determining the HBsAg in human serum ranged from 2.21% to 6.14% (Table 4). The analytical recovery rate ranged from 89.62% to 128.00% (Table 5).

**Table 3:** Functional sensitivity of BC-EDTA/Eu^3+^-nanosphere.

Concentration of HBsAg (μg/L)	Counts^a^	S/N
1.25	12408.667	19.10
0.63	8504.667	13.09
0.31	3175.333	4.89
0.20	2460.333	3.79
0.16	2077.000	3.20
0.08	1394.000	2.15
0.04	1031.000	1.59
0.02	865.667	1.33
0.01	695.667	1.07
0.00	649.667	1.00

^a^ Mean value of net fluorescence counts (n = 10).

**Table 4:** Analytical precision of determination of HBsAg in human serum (n = 20).

Mean value of detected concentration (μg/L)	SD	CV (%)
40.66	2.0051571	4.93%
16.91	1.3407218	7.93%
0.60	0.0561905	9.32%

**Table 5:** Analytical recovery rate of determination of HBsAg in human serum (n = 10).

Basic concentration (μg/L)	Added concentration (μg/L)	Founded concentration (μg/L)	CV (%)	Analytical recovery rate (%)
0	1.00	1.02	6.25	101.90
	5.00	4.48	9.18	89.62
10.00	1.00	0.97	2.20	97.00
	5.00	6.40	2.78	128.00
50.00	5.00	5.62	2.54	112.36
	10.00	11.65	2.41	116.54

**Fig 10:**
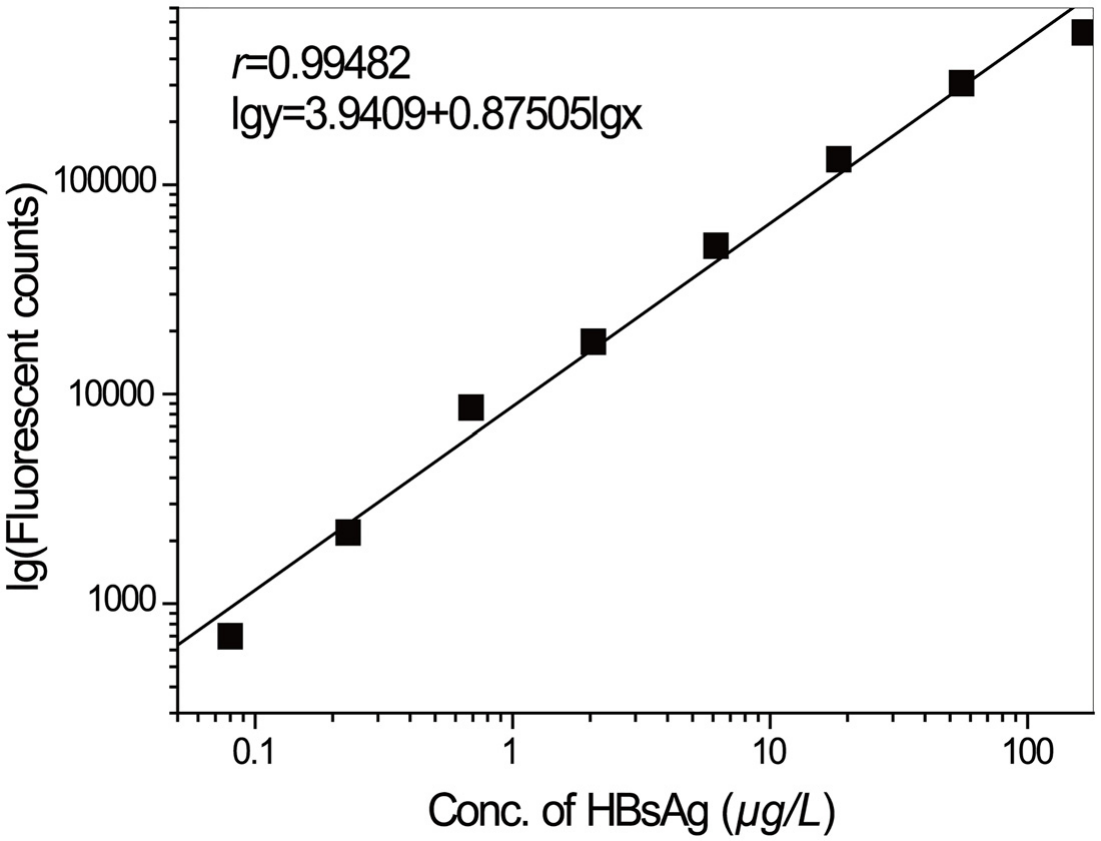
Calibration curves for HBsAg.

The linear range of the nanosphere-based TrFIA of HBsAg was 0.08–166.67 μg/L (Fig 10) which was superior to commercial DELFIA reagents (0.2-145ng/mL). In contrast, the linear range of ELISA was 0.2–5.0 μg/L with a detection limit of 0.2 μg/L.

**Fig 11:**
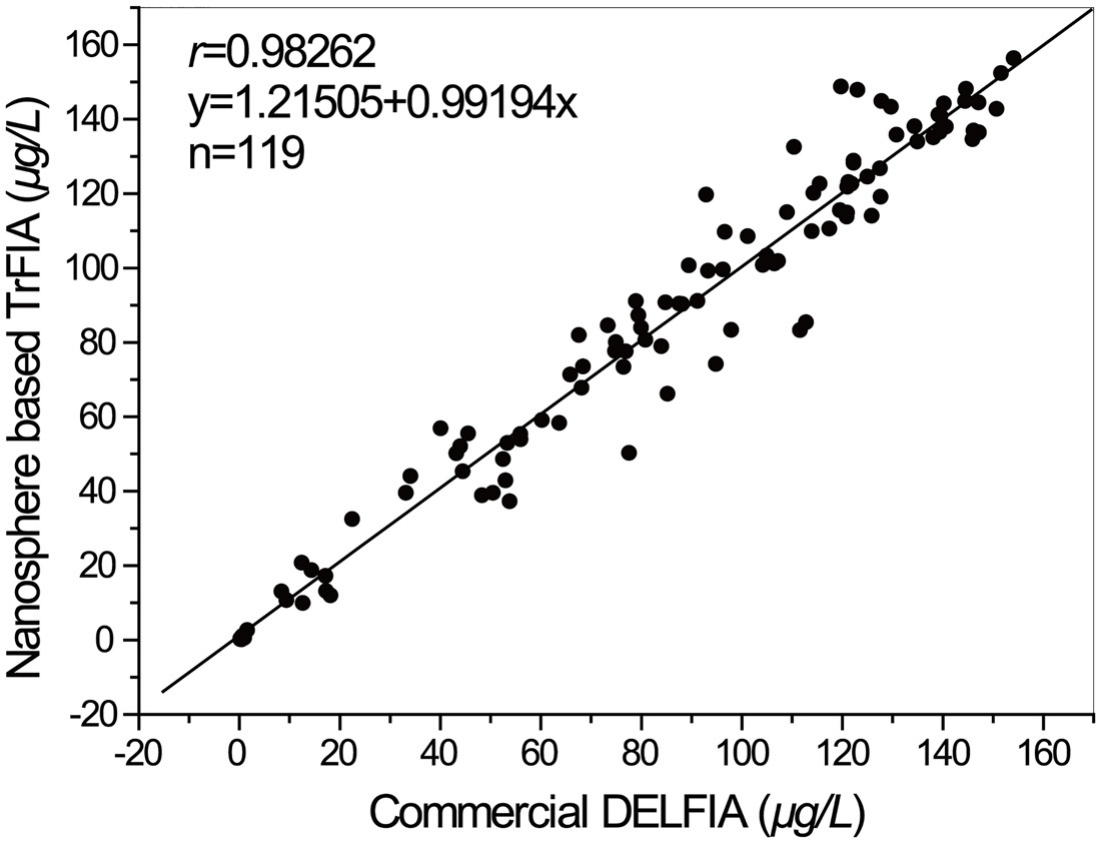
Regression analysis result of BC-EDTA/Eu^3+^-nanosphere based TrFIA and DELFIA.

In the contrast study, 230 human serum samples were detected by the BC-EDTA/Eu^3+^- nanosphere based TrFIA reagent and by the DELFIA reagent. Both of these two methods’ cutoff value was defined as ≥0.2μg/L. Coincidence rate of qualitative analyses of these two methods is 100%. For the 119 positive samples, the correlation coefficient between the BC-EDTA/Eu^3+^-nanosphere based TrFIA and the commercial DELFIA reagents was *r* = 0.98262(Fig 11), results showed that there is a great correlation between these two methods. Result of the HBsAg reference panel also meets our expectation (data not shown).

## Conclusion and Discussion

Our results indicate that the novel europium ligand BC-EDTA is suitable for TrFIA as demonstrated by its excellent water solubility. ESI-MS, Infrared Absorption Spectra, Ultraviolet-Visible (UV) Absorption Spectrum, and H^1^NMR results all showed successful synthesis of the ligand.

BC-EDTA/Eu^3+^ was covalently coated onto the surface of a silica nanosphere. Transmission electron microscope images showed that the fluorescent nanoshpere had no significant visual difference compared with bare silica nanosphere. Taking advantage of the signal amplification effect of the nanosphere, the BC-EDTA/Eu^3+^-nanosphere emitted intense fluorescence when excited by ultraviolet light. The fluorescence lifetime of the BC-EDTA/Eu^3+^-nanosphere is a marginally longer than the free one, which suggests that immobilization can improve the fluorescence lifetime of chelate. Maximum excitation wavelength changed significantly when BC-EDTA reacted with EDC and NHS. This suggests that the phenomeon of maximum excitation shift can be applied in other applications, such as transfer wavelength detection. We plan to explore this area in the future.

Free amino groups on the surface of the BC-EDTA/Eu^3+^-nanosphere makes antibodies can covalently conjugated with the nanosphere. All of these characteristics showed that the BC-EDTA/Eu^3+^-nanosphere can be applied in TrFIA. The procedure for the synthesis of BC-EDTA described here is simple, and the process of preparing a fluorescent nanosphere and an antibody-fluorescent nanosphere complex is simple and easily controllable. The complex can be purified by centrifugation, which is also easy to operate and control.

The antibody-fluorescent nanosphere complex solution emitted an intense and stable signal, and can be applied in the double antibody sandwich method to detect HBsAg. The results showed that the nanosphere based TrFIA system has a high sensitivity (0.0037μg/L), wide linear range (0.08–166.67μg/L, *r* = 0.99482), high recovery rate (89.62–128.00%), and low CV (2.21–6.14%). Benefits of nanosphere based TrFIA include good correlation with commercial DELFIA reagents, ease of use, and decreased Eu^3+^ contamination from the environment. Because of the low background, the BC-EDTA/Eu^3+^-nanosphere based TrFIA has higher sensitivity than the commercial DELFIA reagents. Demonstrated that the novel ligand BC-EDTA and the fluorescent nanosphere described here had very good application prospects in clinical examinations.
